# Enantioselective Molecular Detection by Surface Enhanced Raman Scattering at Chiral Gold Helicoids on Grating Surfaces

**DOI:** 10.1021/acsami.4c09301

**Published:** 2024-09-03

**Authors:** Anastasiia Skvortsova, Jeong Hyun Han, Andrea Tosovska, Polina Bainova, Ryeong Myeong Kim, Vasilii Burtsev, Mariia Erzina, Premysl Fitl, Marie Urbanova, Vaclav Svorcik, In Han Ha, Ki Tae Nam, Oleksiy Lyutakov

**Affiliations:** † Department of Solid State Engineering, University of Chemistry and Technology, Prague 16628, Czech Republic; ‡ Department of Materials Science and Engineering, Seoul National University, Seoul 08826, Republic of Korea; § Department of Physics and Measurements, University of Chemistry and Technology, Prague 16628, Czech Republic

**Keywords:** enantioselective detection, chiral gold nanoparticles, plasmon coupling, SERS, naproxen

## Abstract

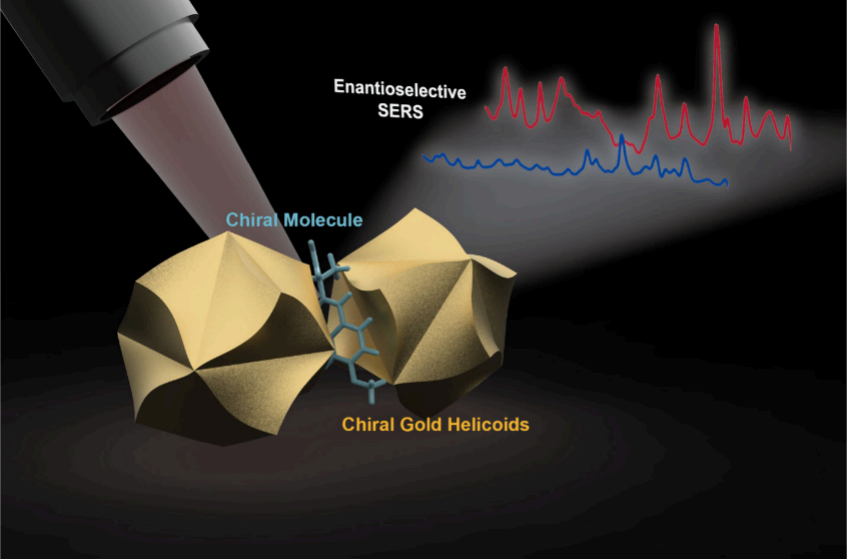

Distinct advantages of surface enhanced Raman scattering (SERS) in molecular detection can benefit the enantioselective discrimination of specific molecular configurations. However, many of the recent methods still lack versatility and require customized anchors to chemically interact with the studied analyte. In this work, we propose the utilization of helicoid-shaped chiral gold nanoparticles arranged in an ordered array on a gold grating surface for enantioselective SERS recognition. This arrangement ensured a homogeneous distribution of chiral plasmonic hot spots and facilitated the enhancement of the SERS response of targeted analytes through plasmon coupling between gold helicoid multimers (formed in the grating valleys) and adjacent regions of the gold grating. Naproxen enantiomers (*R*(+) and *S*(−)) were employed as model compounds, revealing a clear dependence of their SERS response on the chirality of the gold helicoids. Additionally, propranolol and penicillamine enantiomers were used to validate the universality of the proposed approach. Finally, numerical simulations were conducted to elucidate the roles of intensified local electric field and optical helicity density on the SERS signal intensity and on the chirality of the nanoparticles and enantiomers. Unlike previously reported methods, our approach relies on the excitation of a chiral plasmonic near-field and its interaction with the chiral environment of analyte molecules, obviating the need for the enantioselective entrapment of targeted molecules. Moreover, our method is not limited to specific analyte classes and can be applied to a broad range of chiral molecules.

## Introduction

The chiral nature of certain organic molecules holds significant importance in chemistry and biochemistry, necessitating consideration across various practical applications.^1^ Therefore, a key concept for analytical and bioanalytical chemistry is the capability of enantioselective detection of molecular structures, i.e., recognition between molecules with the same chemical composition but opposite absolute configuration.^2,3^ While enantioselective chromatography stands as a conventional method for chirality recognition, alternative approaches have been proposed to streamline enantioselective detection, making it faster and more dependable.^4−6^ Examples include enantioselective electrochemistry, fluorescence, and similar methodologies employing diverse chirality transducers/readers.^7−12^ However, many of these methods rely on the immobilization of specific surface anchors (or their surface imprinting) and their tailored interaction with organic enantiomers.^13−16^ Consequently, the majority of these approaches target specific analytes or families of analytes sharing similar chemical structures, limiting their universality.

Plasmon-active nanostructures warrant special consideration in the detection field, given that their application enables a significant enhancement of the inherently weak Raman signal, pushing toward the absolute detection limit, or the recognition of single molecules (with utilization of so-called SERS, surface enhanced Raman spectroscopy).^17,18^ In turn, enantioselective molecule entrapment on SERS-active surfaces, powered by appropriate surface functionality, facilitates the acquisition of structural and optical conformation-related insights of the targeted analyte, even when dealing with low analyte concentrations or low volumes.^19−22^ Nevertheless, similarly to previous approaches, such detection methodologies are constrained by specific surface–analyte interactions and thus cannot be regarded as universally applicable.^22−25^

In this work, we propose the utilization of gold helicoids for universal SERS-based enantioselective detection. Unlike conventional plasmon-active structures (or chiral ones, formed via self-assembly or templating techniques^26−28^), gold helicoids can support the excitation of a chiral plasmonic near field, confined to the vicinity of the enantiomeric molecule(s).^29−31^ We assume that the intensity of the plasmon generated on the surface of gold helicoids (or within hot spots formed between coupled helicoids) should correlate with the chirality of their environment. Oppositely, the SERS response of the environment depends on both the intensity of the excited plasmon and the local values of the plasmon-related electric field. In essence, the chirality of the environment dictates the efficacy of plasmon excitation, while the efficiency of plasmon excitation (specifically in the case of gold helicoids supporting the chiral plasmonic near field) determines the intensity of the SERS signal originating from the environment. Indeed, such an approach is not confined to specific organic enantiomers but can be extended to a broad spectrum of chiral molecules, rendering it universal. This assumption is subsequently verified and utilized in our study.

## Results and Discussion

### Main Experimental Concept

**Figure 1:**
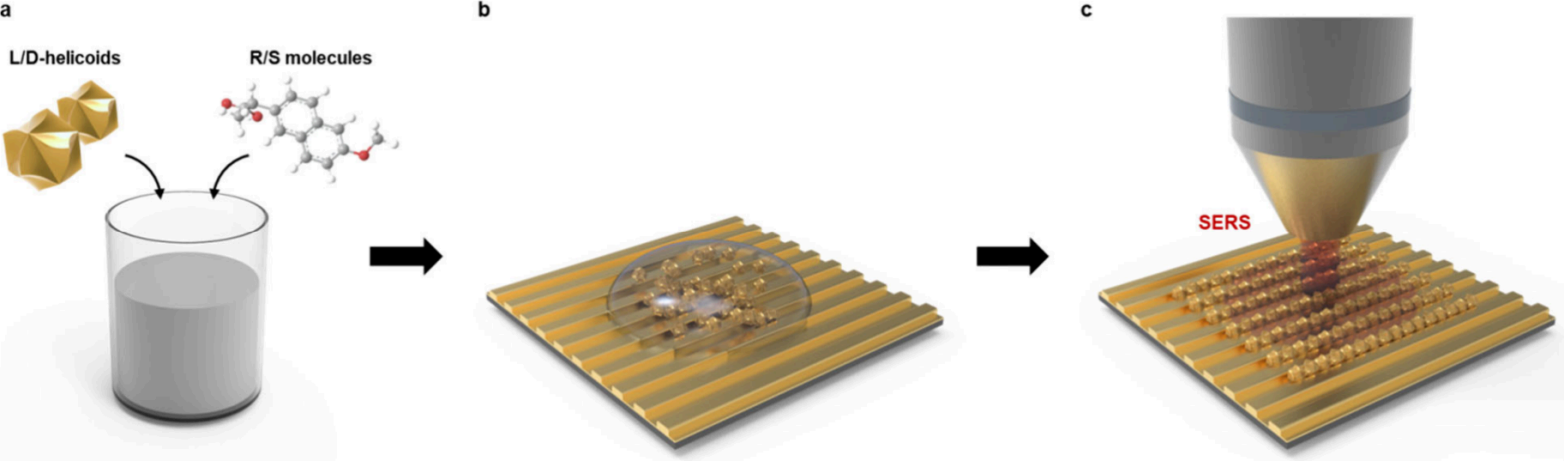
Schematic representation of proposed approach: creation of chiral nanoparticles, their mixing with chiral analyte (a), deposition on plasmon active periodical grating (b), and subsequent SERS measurements (c).

Our main experimental approach is schematically presented in Figure 1. First, the chiral gold nanoparticles (helicoids) were synthesized using the previously reported procedure.^29,30^ As mentioned before, utilization of such nanoparticles enables the excitation of chiral near-fields through the chirality encoded in the framework of each single nanoparticle, primarily dictated by its shape.^31^ Subsequently, the left- or right-handed helicoids were mixed with the targeted analyte and deposited on the surface of a plasmon-active gold grating. The deposition process was optimized to achieve an ordered distribution of gold helicoids on the grating surface, forming densely packed multimer “strips”. In this context, the gold grating ensured both the ordering of nanoparticles into strip formations with well-defined distances between helicoids and the excitation of surface plasmon polariton (SPP) waves, aimed at dispersing the plasmon intensity across the sample surface uniformly (to ensure homogeneous distribution of the SERS signal). Additionally, coupling between the local surface plasmon (LSP) from chiral nanoparticles and the SPPs generated by the periodic grating enhanced the local electric field strength and increased the SERS signal.

In our experiments, we used biomedically relevant naproxen enantiomers as a model compound. The samples with gold helicoids and enantiomers were subjected to SERS measurements, focusing on the enantiomer signal variation depending on the interplay of organic molecules and helicoids handedness. Finally, the universality of our approach was demonstrated on alternative organic molecules, propranolol and penicillamine enantiomers (both compounds have different biochemical impacts, determined by their absolute configuration).

### Au Helicoids Characterization

**Figure 2:**
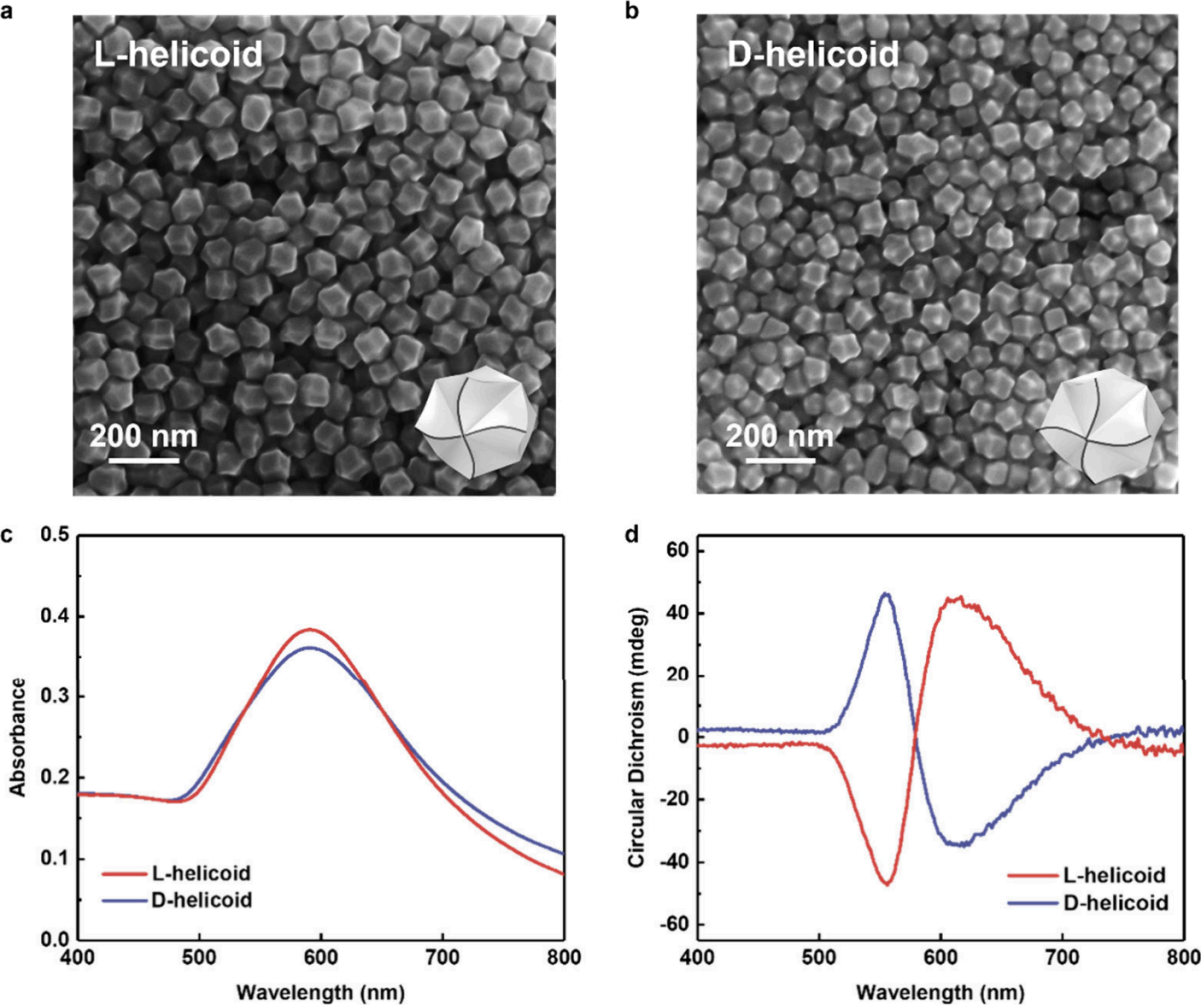
Structures characterization: (a) SEM of l-helicoids, (b) SEM of d-helicoids, and (c) UV–vis and (d) CD spectra of helicoids.

The structure of the helicoids was confirmed using SEM measurements performed at different magnifications (Figures 2a and 2b). SEM images reveal the homogeneous size distribution of helicoids, which correlates with DLS results (Figure S1, nanoparticle size of approximately 110 nm). In addition, created gold nanoparticles have a specific morphological feature, with a tendency to be shaped at left- or right-handed depending on the cysteine handedness used during the procedure (see inset in Figures 2a vs 2b). Such helicoid morphology is commonly related to the appearance of optical chirality at characteristic wavelengths of the plasmon absorption band. In our case, the plasmon absorption bands, measured in nanoparticle aqueous suspensions, were located between 500 and 750 nm for both helicoid types (Figure 2c). At these wavelengths, we also observed the appearance of optical chirality, evident as an apparent difference in the absorption of left and right circularly polarized light (Figure 2d). Finally, the measured circular dichroism (CD) signal was symmetrical about the *x*-axis, which indicates the opposite and “symmetrical” chirality of a left- or right-handed nanoparticles.

### Creation of Ordered Au Helicoids Array and SERS Substrates Optimization

**Figure 3:**
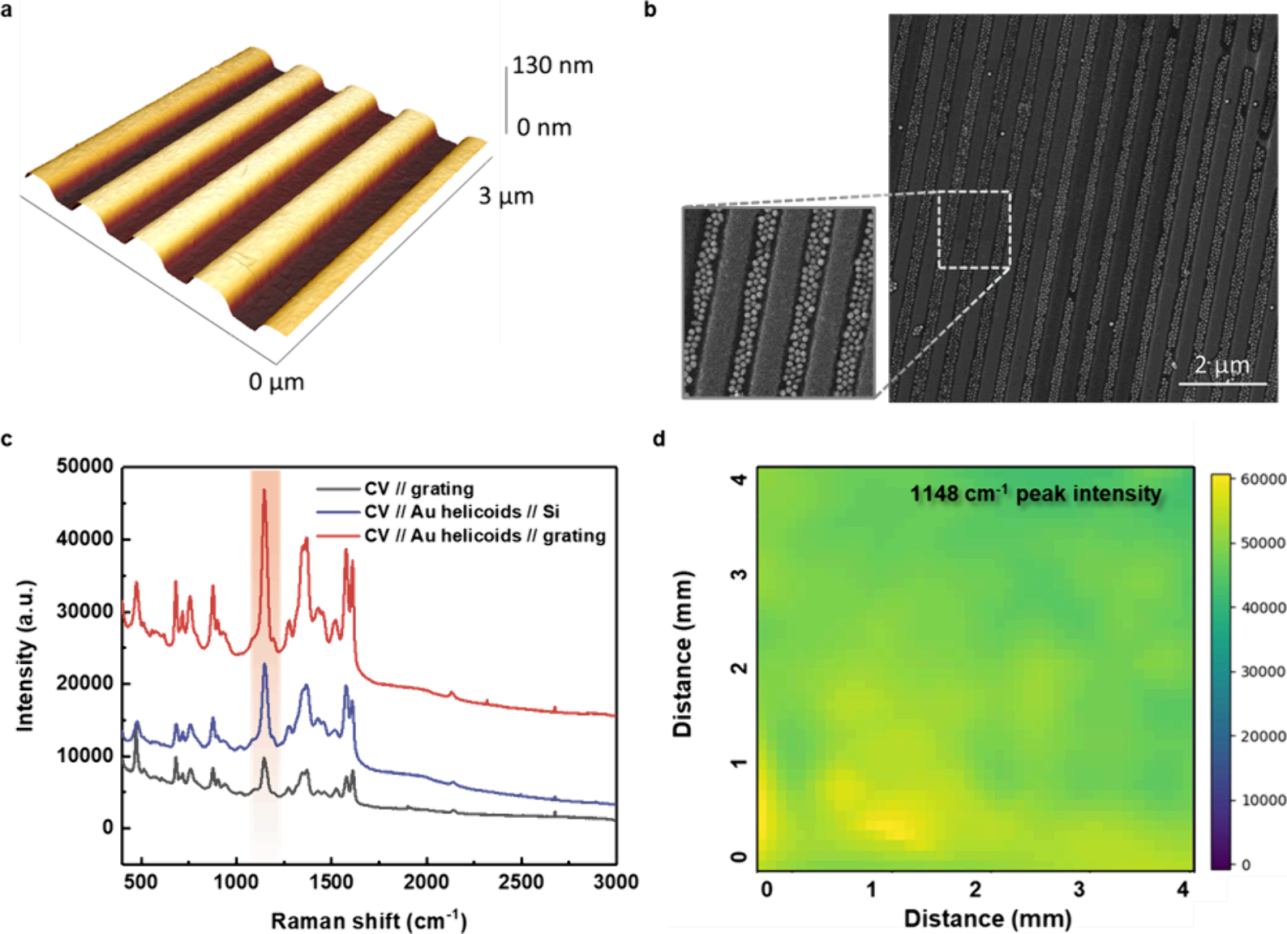
(a) AFM measured surface morphology of pristine gold grating; (b) SEM image of gold grating with helicoids (inset shows the SEM image with higher magnification); (c) SERS spectra of CV on different substrates (gold grating, helicoids random array, ordered helicoids array on gold grating surface); and (d) spatial distribution of the intensity of characteristic CV peak (1148 cm^–1^) across helicoids//grating surface.

In the next step, the helicoids were deposited on the Au grating surface. We used the plasmon active grating with periodical morphology (Figures 3a and S2), able to support the excitation and propagation of the SPP wave as confirmed by UV–vis measurements (Figure S3, plasmon absorption band position between 650 and 780 nm). The deposition conditions (solvent, nanoparticle concentration, and methods of deposition) were optimized to reach the formation of ordered, closely packed Au helicoids, located in the grating valleys (Figure S4 shows an example of unsatisfactory helicoids distribution). As a result of optimization, we obtained periodically ordered arrays of helicoids with homogeneous distribution of gaps between the nanoparticles (see Figure 3 and the corresponding insert). We note that this high-density packing was greatly benefited by the unique basal rhombic dodecahedral shape of the helicoids. It was expected that such structures could ensure the homogeneous distribution of plasmonic hot spots in the space between Au helicoids, which in turn could compensate the typical inhomogeneity of SERS signals, measured on the surface of nonordered arrays of plasmonic nanoparticles. The excitation of the traveled plasmon wave on the grating surface will provide additional triggering of hot spots between helicoids and increase in this way the intensity of the SERS signal. Finally, in terms of the chirality of the nanogap, the high symmetry of 432 Au helicoids will enable the formation of a field with consistent chirality in every individual nanogap.

**Figure 4:**
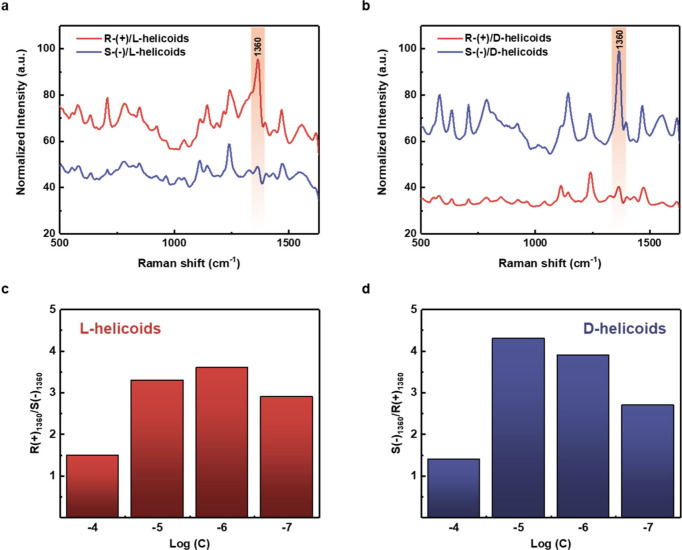
(a and b) Averaged SERS spectra of *S*(−)- or *R*(+)-naproxen enantiomers (deposited from 10^–6^ M solutions), measured with utilization of l- or d-helicoids//grating samples (spectra were measured at 100 different spots across a macroscopic sample area of 5 × 5 mm^2^ and deviation between spectra was less than 3.5%). (c and d) Ratios (*R*(+)/*S*(−) or vice versa) of characteristic enantiomer peak (1360 cm^–1^) intensity, measured on l- or d-helicoids//grating samples as a function of initial enantiomer concentrations.

In order to verify the distribution of the SERS signal over the sample surface, a common SERS probe (crystal violet, CV, initial concentration in methanol 10^–6^ M) was used. CV was deposited on the surface of three samples: gold grating without helicoids, nonordered (from long distance point of view) array of helicoids prepared on a flat silicon substrate, and an optimized sample–gold grating with helicoids array. SERS spectra (obtained in the “mapping” regime at 785 nm excitation wavelength and presented as an averaged value from 60 SERS spectra) are presented in Figure 3c. In all cases we observed the appearance of apparent CV-related peaks despite low analyte surface concentration, indicating the SERS enhancement (CV does not absorb the excitation wavelengths;Figure S5, absorption band position between 500 and 650 nm). So, the response should be attributed to a pure SERS, not to some side phenomena (e.g., resonant Raman effects). The peak intensities also indicate the efficiency of SERS enhancement, which corresponds to the order grating < helicoids < helicoids//grating. In the later case the higher SERS enhancement should be attributed to the plasmon coupling between plasmonic waves, excited on the Au grating surface and local plasmonic hot spots between Au helicoids. In turn, Figure 3d shows the distribution of the characteristic 1148 cm^–1^ peak intensity along the Au grating/gold helicoid sample surface. As is evident, the homogeneous peak intensity was achieved across the whole sample surface (4 × 4 mm^2^) thanks to the well-ordered nature of the sample surface (a similar convergence of SERS signals was observed in the case of measurements between individual substrates; Figure S6). For comparison, we also provide similar SERS maps, measured on Au grating and on a helicoids array (Figure S7). Obviously, the SERS signal obtained on the grating surface is homogeneous too, but the intensity is lower than in the case of the helicoids array deposited on a nonplasmonic Si substrate. However, apparent variations of the signal intensity in the case of the helicoids array were observed. The structure combining Au grating with deposited helicoids allows us to achieve a synergy of all advantageous effects mentioned above (Figures 4c and S7). In this case, the SERS signal intensity was enhanced (compared to grating cases), while the signal homogeneity was still comparable to the pristine grating. Therefore, the specific morphology presented in Figure 3 allowed us to achieve high SERS enhancement and proper signal distribution, which are the key parameters for subsequently demonstrating enantioselective SERS detection.

### Universal Enantioselective SERS Detection

In the next step, we proceeded with enantioselective SERS discrimination. As a first model compound, we used naproxen enantiomers. First, Raman and SERS spectra of naproxen enantiomers (measured either in powder form or after the deposition on Au grating surface) showed no difference (Figure S8). The typical SERS spectra of *R*(+)- and *S*(−)-naproxen enantiomers, measured on l- and d-helicoids/grating samples, are presented in Figures 4a and 4b. In this case, we used the initial naproxen enantiomer concentration of 10^–5^ M, and the SERS spectra contain information from naproxen molecules and also from residual CTAB, which is used as a surfactant during the preparation of nanoparticles (Figure S9 shows that CTAB molecules were partially removed during the interaction of Au helicoids with the targeted analyte solution). We also did not observe any SERS response of the cysteine residual molecules^32,33^ (because their lower concentration in the solution used for helicoids preparation and the intrinsically small Raman scattering cross section of cysteine). The peaks at 1625, 1547, 1360, and 708 cm^–1^^34^ belong to the naproxen enantiomer(s) and can be attributed to C=O stretching vibrations, aromatic ring stretching, COO^–^ stretching, and out-of-plane bending vibrations of the aromatic ring (from their relatively high intensities, it is evident that the analyte molecules occupy the plasmonic hot spots between Au helicoids, while the CTAB molecules are partially removed during the sample preparation in the methanol solution). From Figure 4a it is evident that there is an apparent difference in SERS peak intensity (especially the peaks attributed to naproxen). We observed higher intensities of SERS spectra in the case of *R*(+)-naproxen//l-helicoids/grating and *S*(−)-naproxen//d-helicoids/grating combinations, while in the opposite cases the peak intensities were notably lower. In what follows, the 1360 cm^–1^ peak related to the COO^–^ group with great binding affinity to gold facet and located adjacent to the chiral center of molecule was used for estimation of concentration-related decencies to *R*(+)- or *S*(−)-naproxen enantiomer SERS responses.

To highlight the different SERS enhancement, the enantiomer band ratio, measured on l-helicoids/grating or d-helicoids/grating surfaces, is plotted as a function of enantiomer(s) concentration in Figures 4c and 4d (the absolute values of peak intensities are plotted against the initial enantiomer concentration in Figure S10; evident nonlinear dependence indicates the deviation of enantiosensitive SERS from “common” SERS). As is evident, the apparent differences between band intensities (up to 3–4 times) were reached for 10^–5^–10^–6^ M naproxen concentrations. At higher or lower enantiomer concentrations, less pronounced ratios of band intensities are observed. This can be attributed to the insufficient amount of naproxen molecules, which are located in the chiral plasmonic hot spot in the case of lower analyte concentrations. For higher concentrations, the obtained results can be explained in the light of the subsequently performed numerical calculation and the gradual increase of κ (chirality parameter).

In general, from Figure 4 we can conclude that the naproxen enantiomers produce different SERS signals (in terms of SERS enhancement) as a function of the chirality of the near plasmonic field. Since naproxen does not interact specifically with helicoid surfaces, a similar phenomenon could be also expected in the case of other chiral organic molecules. To check this assumption, we performed additional enantioselective discrimination of propanol and penicillamine enantiomers (both can be considered as medically relevant compounds with molecular configuration-determined functionality^35,36^). The results are presented in Figures S11 and S12 (the spectra represent an average of 50 measurements at different points on the sample surface). For both propranolol and penicillamine enantiomers, we observed a notable difference in the enantiomeric response depending on the neighboring SERS substrate: a gold grating with deposited l- or d-helicoids. Specifically, the disparity in enantiomer SERS responses, characterized by the more pronounced peak intensity, fell within the range of 3–3.5 times. This observation leads us to conclude that the proposed approach is versatile, i.e., not limited to a particular organic molecule or class of organic compounds. Thus, it is able to distinguish enantiomeric chirality independently of their physicochemical interaction with the surface of helicoids (or enantioselective entrapment on SERS active surface), unlike traditional methods used in the field of enantioselective SERS. In turn, a series of control experiments were performed involving the nanoparticle array deposited on a silicon surface (i.e., without the neighboring grating). In this case, we observed the significant deviations of SERS signal intensity measured at different spots on the Au helicoid aggregates. However, averaging of SERS spectra measured on different spots allows us to find a difference between organic enantiomers (Figure S13). In contrast, the utilization of spherical Au nanoparticles, deposited on the Au grating surface, does not lead to any difference in the peak intensities of the opposite enantiomers in SERS spectra (Figure S14). Finally, the SERS measurements performed with the utilization of different excitation wavelengths, corresponding to the light absorption by helicoids (not by Au grating), indicate that the utilization of an alternative wavelength (e.g., 633 nm) also allows for the enantioselective discrimination (Figure S15). It is noteworthy that all SERS experiments were conducted using a standard Raman spectrometer with nonpolarized light. Our approach does not necessitate the use of specific (circular) polarization of laser excitation light or the implementation of polarization-sensitive detectors. Even with the conversion of nonpolarized light into a chiral distribution of the plasmonic field on the surface of gold helicoids we achieved enantioselective discrimination of organic molecules. These circumstances increase the versatility of our method markedly. It should also be noted that in the close-to-real situation, organic enantiomers can be presented in a mixed form. Our approach is also suitable even for the analysis of these samples: the enantiomers mixture can be measured on l- and d-helicoids/grating SERS substrates (optionally, on a nonchiral Au grating surface to determine the “absolute” analyte concentration) and the enantiomers ratio can be subsequently determined using the known concentration and relative intensity of characteristic SERS peak(s), obtained on substrates with different chirality.

### Numerical Simulation: Estimation of Enantioselective SERS Detection Mechanism

Based on our experimental findings, we conducted a series of numerical simulations to elucidate the origin of exceptional enantioselective SERS signals. Our results indicate that these signals arise not only from the local electric field enhancement facilitated by closely packed Au helicoids but also from selective molecular excitation driven by optical chirality. Conventionally, the SERS enhancement factor is recognized to be proportional to the fourth power of the local electric field intensity (|**E**|^4^) while the enhancement of Raman scattered signals and the molecular excitation are each proportional only to |**E**|^2^.^37,38^ Molecular excitation can be estimated as follows. Since the electric dipole moment of a molecule with electric polarizability α is given by **p** = α**E**, the energy acquired during the excitation process is . Taking into account the chirality when a chiral molecule interacts with an electromagnetic field, the involvement of chiral polarizability *G* (i.e., chirality parameter κ), alongside electric polarizability α and magnetic polarizability β leads to the following induction of electric and magnetic dipole moments: **p** = α**E** + *iG***H** and **m** = β**H** – *iG***E** ≈ −*iG***E** (negligible contribution of magnetic polarizability β can be ommited). Then the energy acquired by the chiral molecule expressed as  is influenced by both the local electric field intensity |**E**|^2^ and the optical helicity density , which characterizes the helicity and strength of the chiral electromagnetic wave.^39−43^ The differential excitation depending on the molecule’s chirality and the optical chirality of the field leads to enantioselective SERS enhancement.^44^

**Figure 5:**
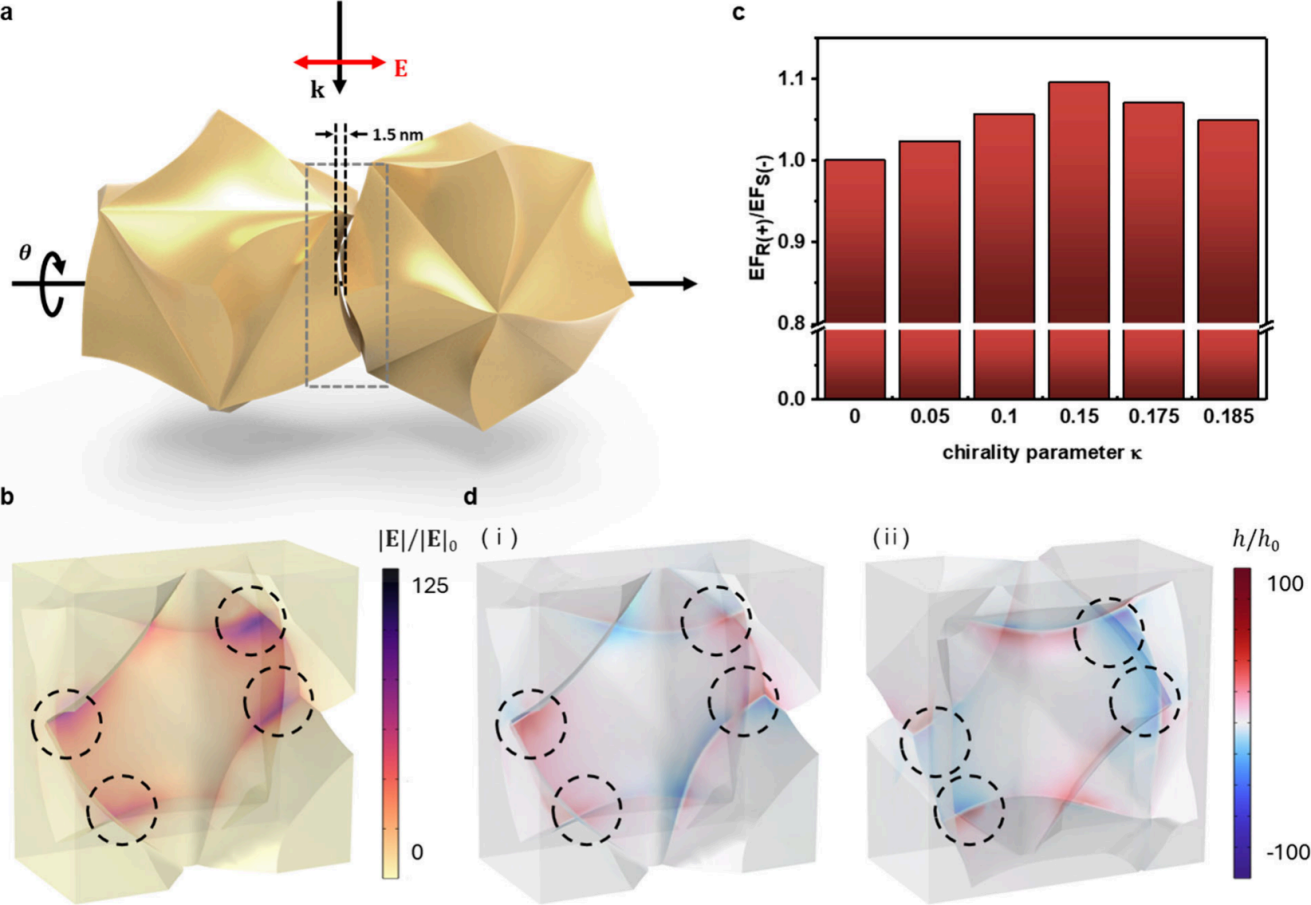
(a) Configuration of the l-helicoid dimer for numerical simulations. The nanogap spacing is set to be 1.5 nm considering the molecular size of naproxen, and the relative rotation angle θ was controlled to be 30, 45, and 60 degrees. (b) Spatial profiles of electric field (|**E**|^2^) at the nanogap indicated with gray box in (a) of the l-helicoid dimer for θ = 45 deg. (c) Enhancement factor depending on the chirality parameter κ of the surrounding medium calculated by volume integration of electric field. (d) Spatial profiles of optical helicity density *h* at the nanogap of (i) l-helicoid and (ii) d-helicoid dimers for θ = 45 deg.

To clarify the role of both electric field **E** and optical helicity density *h* enhancement in determining the enantioselective differences in SERS signal intensity, we try to model the chiral environment near the particles accurately. We adapted the constitutive equation within the wave optics module of the COMSOL Multiphysics numerical simulator.^45−47^ This modification allowed us to incorporate both the refractive index *n* and the chirality parameter κ of the medium. The 3D model of the helicoid was constructed based on the SEM image (Figure 2a) and the previously reported crystallographic interpretation (Figure S16).^30^ Using the dimensions of naproxen molecules as a reference one (1.41 nm), we constructed an l-helicoid dimer with a 15°, 30°, and 45° relative rotation angle and 1.5 nm spacing and analyzed the electric field distribution at their nanogap (Figure 5a). Our simulations revealed the formation of intense electric field hotspots along the concave boundary of the dimeric gap, consistent with established factors contributing to the SERS enhancement (Figure 5b). The strong localization of the electric field in such chiral geometries suggests an amplification of the enantioselectivity in interactions with chiral molecules, a phenomenon supported by our calculations of optical helicity density *h*. Furthermore, as displayed in Figure 5c, the trend of the ratio of volume-integrated electric field (i.e., enhancement factor) for opposite chirality of media (i.e., the sign of chirality parameter κ) increasing and then decreasing as the chirality of media increases is coherent with experimental observations for peak intensity ratios between *R*(+)/*S*(−)-naproxen displayed in Figures 4c and 4d. Analyzing the electric field profiles, we observed that when chirality parameter κ surpasses a certain threshold, the relative impact of electric field outside the dimeric hotspot increases (Figure S17). This finding suggests that the diminishing enantiomeric ratio of the signal may result from the increased influence of chiral molecules beyond the dimeric nanogap.

The calculation of the optical helicity density *h* profile provides a robust basis for understanding the origin of the enantioselective SERS signals. Our findings, depicted in Figure 5d, reveal that despite linear polarization, optical helicity density *h* values at the dimeric nanogap can be locally enhanced up to 100-fold compared to propagating circularly polarized plane waves in free space. This underscores the unique advantage of our platform in imparting high enantiopreference without necessitating circular polarization formation at the light source level, as demonstrated experimentally too. Also, we confirmed that when the chirality of the particles is opposite, i.e., simulated with the dimer of d-helicoid, the sign of the condensed optical helicity density *h* also becomes opposite. As can be seen from the above equation on chiral molecular excitation , the sign of calculated optical helicity density  clearly directly affects the selective signal enhancement pairs (*R*(+)-naproxen//l-helicoid and *S*(−)-naproxen//d-helicoid) between the chirality of particles and molecules. Another notable aspect is the consistent sign of local optical helicity density *h* at the electric field hotspots (Figure 5b), which contributes to a uniform enhancement of nonvanishing enantioselective signals.^41,48^ This consistent behavior, unaffected by the assembly angle of the helicoid dimer (Figure S18), is attributed to the maintenance of 432 symmetry in nanoparticle facets, leading to the formation of electric field hotspots with uniform optical helicity density *h*. Consequently, this results in strong and consistent enantioselective signals, unaffected by random molecular distribution in regards to helicoid(s) orientation.

## Conclusion

In this study, we propose a versatile SERS-based approach for the enantioselective recognition of organic compounds. Our strategy involves arranging chiral gold helicoids (either left- or right-handed) into ordered strip structures on the surface of a gold grating. These gold helicoids can support the excitation of chiral plasmonic near-fields, even when illuminated with nonpolarized light. We hypothesized that the interaction of the chiral plasmonic near-field with organic enantiomers contributes to SERS spectra of different intensities, enabling universal enantioselective discrimination. Additionally, the ordered array of gold helicoids facilitates the homogeneous distribution of plasmonic hot spots and the resulting SERS signals, enhancing the differentiation between the SERS responses of organic enantiomers based on the handedness of gold helicoids. Furthermore, the use of a gold grating as a substrate promotes plasmonic coupling, leading to further amplification of the SERS signals from analytes. Our approach was validated using enantiomers of naproxen, propranolol, and penicillamine, for which clear differences in SERS signals were observed in dependence on the handedness of gold helicoids (i.e., the chirality of the ordered gold nanoparticles). We highlight the critical roles of electric fields and optical chirality in SERS enhancement and chiral discrimination by solving the constitutive equation governing the electromagnetic dipole excitation of chiral molecules. Through numerical analysis, we interpret the coenhancement of electric fields and uniform-sign optical helicity density as the underlying mechanism of our system, which can serve as a universally applicable enantioselective Raman signal enhancement platform. Created SERS substrates can be employed to assess the chirality of various organic molecules at low concentrations. Moreover, the experimental procedure can be conducted using standard Raman spectrometers, eliminating the need for a chiral light source or polarization-sensitive detectors.

## Experimental Section

### Materials

Hexadecyltrimethylammonium bromide (≥98.0%, Sigma-Aldrich), l-ascorbic acid (99.0%, Sigma-Aldrich), d-cysteine (≥99.0%, Sigma-Aldrich), l-cysteine (98.5%, Sigma-Aldrich), hydrogen tetrachloroaurate(III) trihydrate (99.99%, ABCR), water for chromatography (Sigma-Aldrich), sodium borohydride (99.0%, Sigma-Aldrich), crystal violet (≥90.0%, Sigma-Aldrich), ethyl alcohol (96.0%, Sigma- Aldrich), *S*(+)-6-methoxy-α-methyl-2-maphthaleneacetic acid (98.0%, Sigma-Aldrich), *R*(+)-propranolol hydrochloride, and *S*(−)-propranolol hydrochloride were used.

### Sample Preparation

#### Synthesis of Chiral Nanoparticles

Initially, cubic seeds were prepared as reported previously.^49,50^ A growth solution was then created by mixing 0.8 mL of a 100 mM CTAB solution with 0.2 mL of a 10 mM gold chloride trihydrate solution in 3.95 mL of deionized water. Then, 0.475 mL of a 100 mM l-ascorbic acid solution was injected into the growth solution to reduce Au^3+^ to Au^+^. Further, formation of chiral nanoparticles was carried out according to the previously published methodology, albeit with some modifications. Then, 0.05 mL of cubic seeds (Figure S19, nanoparticle size: approximately 40 nm) were added to the growth solution, and after a 20 min incubation period, 0.005 mL of 0.1 mM l- or d-cysteine solution was added. The growth solution was incubated at 30 °C for 1 h, during which time its color changed from pink to blue. The solution was subjected to two cycles of centrifugation (4000 rpm, 10 min) and then dispersed in dH_2_O for further characterization.

The Au helicoid deposition procedure was optimized to achieve an ordered distribution of the nanoparticles on the Au grating surface. A gold sputtered grating on the DVD disk (40 mA, 350 s sputtering time) was placed on a strictly horizontal surface in the desiccator at room temperature. The 200 μL of helicoid solution was then mixed with 200 μL of naproxen enantiomers (different concentrations), and the drop was deposited on the grating surface. The samples were then dried in the desiccator. A few alternative ways of Au helicoid deposition were also checked, including spin coating, electrophoretic deposition, or drop casting in a water-vapor-saturated atmosphere or under sample tilting by some angle with respect to the Au grating orientation, but suitable results (in terms of nanoparticles spontaneous self-assembling on the Au grating surface) were obtained in a simpler way, described above.

#### Preparation of Spherical Au Nanoparticles

Spherical gold nanoparticles (AuNPs) were synthesized according to a previously published methodology.^51^ Briefly, 50 mL of 5 mM aqueous HAuCl_4_ solution was brought to boiling, and 15 mL of a 40 mM aqueous solution of sodium citrate tribasic dihydrate was then rapidly added to the boiling solution. The formed AuNPs were washed thoroughly using three precipitation–dispersion cycles in deionized water. For further use, the resulting precipitate was redispersed in methanol and mixed with a targeted analyte solution.

### Measurement Techniques

#### Surface Enhanced Raman Spectroscopy (SERS)

SERS was performed on portable ProRaman-L spectrometer with a 785 nm excitation wavelength. Measurement conditions were 30 mW laser power, 100 s accumulation time, and 1 average. Spectral maps were taken on a 300 × 300 μm^2^ surface area with a step of 30 μm between measurement spots. Spectral resolution of 2 cm^–1^ in the 3000–150 cm^–1^ wavenumber range was used. In the case of control SERS measurements, performed on Au helicoids deposited on a silicon substrate (instead of the Au helicoids//Au grating system), the spectra were collected at 30 randomly chosen spots, and an averaged spectrum is presented. Control SERS measurements at the alternative excitation wavelengths were performed using a Nicolet DXR3 Raman microscope. The parameters of measurements were as follows: 1 mW laser power, 5 s accumulation time, and 25 averages for the 633 nm excitation wavelength and 0.1 mW laser power, 5 s accumulation time, and 25 averages for the 532 nm excitation wavelength.

#### Transmission Electron Microscopy (TEM)

TEM was performed with a JEOL JEM-1010 instrument operated at 80 kV (JEOL, Ltd., Japan). The scanning electron microscopy (SEM) was performed on LYRA3 GMU (Tescan, CR) microscope with an accelerating voltage of 2 kV. Raman (in particular, SERS) spectra were measured on a ProRaman-L spectrometer with a 785 nm excitation wavelength (laser power 40 mW). The dynamic light scattering (DLS) was performed using the Zatesizer Ultra system (Malvern, USA).

The UV–vis absorption spectra were measured using a Lambda 25 UV/vis/NIR spectrometer (PerkinElmer, USA) at a scanning rate of 480 nm min^–1^ in the 300–1000 nm wavelength range. Circular dichroism (CD) spectra were measured on a J-810 spectrometer (Jasco, Japan) with a scanning speed of 100 nm/min, a bandwidth of 1 nm, the standard sensitivity setting, and an integration time of 1 s for each spectral point. Optical rotation measurements were performed with the utilization of a Bellingham polarimeter.

## Data Availability

The data presented in this study are available at https://zenodo.org/records/12749705.

## Supporting Information Available

Experimental details, including materials, samples preparation, measurement techniques, simulation methods; TEM and DLS of Au nanoparticles; SEM image and UV–vis absorption spectrum of the pristine gold grating; example of unsuccessful distribution of Au helicoids; UV–vis absorption spectra of crystal violet; SERS maps of crystal violet; Raman and SERS spectra of pure *R*(+)-naproxen and *S*(−)-naproxen; SERS spectra of l- or d-penicillamine; SERS spectra of *R*- or *S*-propranolol; simulated electric field intensity in the vicinity of a l-helicoid dimer depending on the chirality parameter κ of surrounding medium; and spatial profiles of electric field (|**E**|^2^) and optical helicity density *h* at the nanogap of the l-helicoid dimer (PDF)
