# Supplementary material for: Enantioselective Molecular Detection by Surface Enhanced Raman Scattering at Chiral Gold Helicoids on Grating Surfaces
Source: ACS Appl Mater Interfaces. 2024 Sep 3;16(36):48526–35. doi: 10.1021/acsami.4c09301 (PMC11403552; doi:10.1021/acsami.4c09301)
Supplement: Supplementary file 1 — am4c09301_si_001.pdf [file am4c09301_si_001.pdf]

Supporting Information for:

Enantioselective Molecular Detection by Surface-Enhanced Raman Scattering at Chiral Gold Helicoids on Grating Surface

Anastasiia Skvortsova[a†], Jeong Hyun Han[b†], Andrea Tosovska[a], Polina Bainova[a], Ryeong Myeong Kim[b], Vasilii Burtsev[a], Mariia Erzina[a], Premysl Fitl[c], Marie Urbanova[c], Vaclav Svorcik[a], In Han Ha[b], Ki Tae Nam[b*], Oleksiy Lyutakov[a*]

[a] *Department of Solid State Engineering, University of Chemistry and Technology, 16628 Prague, Czech Republic*

[b] *Seoul National University, Department of Materials Science and Engineering, Seoul National University, Seoul 08826, Republic of Korea*

[c] *Department of Physics and Measurements, University of Chemistry and Technology, 16628 Prague, Czech Republic*

Corresponding authors: lyutakoo@vscht.cz, nkitae@snu.ac.kr

[†] These authors contribute equally

1. Simulation Methods

Electromagnetic simulations

In order to numerically simulate the bi-isotropic behavior of chiral media, the built-in functions of wave optics module in commercial finite element method (FEM)-based Maxwell's equation solver (COMSOL Multiphysics) were modified[1–3]. The constitutive equations for the chiral media are:

$$\mathbf{D} = \varepsilon\varepsilon_0\mathbf{E} + i\kappa/c\mathbf{H}$$

$$\mathbf{B} = \mu\mu_0\mathbf{H} - i\kappa/c\mathbf{E}$$

where **E** and **H** are the electric and magnetic field, respectively, **D** is the electric displacement field, **B** is magnetic induction field, respectively, ε is the relative permittivity, ε_0 is the vacuum permittivity, μ is the relative permeability, μ_0 is the vacuum permeability, and κ is the chirality

parameter of the media. Based on these constitutive equations, the magnetic field **H** with related terms corresponding to electric field **E** can be expressed as follows:

$$\mathbf{H} = (\mu\mu_0)^{-1}\left(\mathbf{B} + i\frac{\kappa}{c}\mathbf{E}\right)$$

$$\frac{\mathrm{d}\mathbf{H}}{\mathrm{dt}} = (\mu\mu_0)^{-1}\left(\frac{\mathrm{d}\mathbf{B}}{\mathrm{dt}} - i\frac{\kappa}{c}\omega\mathbf{E}\right)$$

Reflecting these, the COMSOL equations for the chiral media domain were modified as follows:

ewfd.Dx → epsilon0_const*ewfd.Ex+ewfd.Px-1i/c_const*kappa*ewfd.Hx

ewfd.Dy → epsilon0_const*ewfd.Ey+ewfd.Py-1i/c_const*kappa*ewfd.Hy

ewfd.Dz → epsilon0_const*ewfd.Ez+ewfd.Pz-1i/c_const*kappa*ewfd.Hz

ewfd.Hx → (ewfd.murinvxx*ewfd.Bx+ewfd.murinvxy*ewfd.By+ewfd.murinvxz*ewfd.Bz-1i/c_const*kappa*(ewfd.murinvxx*ewfd.Ex+ewfd.murinvxy*ewfd.Ey+ewfd.murinvxz*ewfd.Ez))/mu0_const

ewfd.Hy → (ewfd.murinvyx*ewfd.Bx+ewfd.murinvyy*ewfd.By+ewfd.murinvyz*ewfd.Bz-1i/c_const*kappa*(ewfd.murinvyx*ewfd.Ex+ewfd.murinvyy*ewfd.Ey+ewfd.murinvyz*ewfd.Ez))/mu0_const

ewfd.Hz→ (ewfd.murinvzx*ewfd.Bx+ewfd.murinvzy*ewfd.By+ewfd.murinvzz*ewfd.Bz-1i/c_const*kappa*(ewfd.murinvzx*ewfd.Ex+ewfd.murinvzy*ewfd.Ey+ewfd.murinvzz*ewfd.Ez))/mu0_const

ewfd.dHdtx → (ewfd.murinvxx*ewfd.dBdtx+ewfd.murinvxy*ewfd.dBdty+ewfd.murinvxz*ewfd.dBdtz-1i*ewfd.iomega/c_const*kappa*(ewfd.murinvxx*ewfd.Ex+ewfd.murinvxy*ewfd.Ey+ewfd.murinvxz*ewfd.Ez))/mu0_const

ewfd.dHdty → (ewfd.murinvyx*ewfd.dBdtx+ewfd.murinvyy*ewfd.dBdty+ewfd.murinvyz*ewfd.dBdtz-1i*ewfd.iomega/c_const*kappa*(ewfd.murinvyx*ewfd.Ex+ewfd.murinvyy*ewfd.Ey+ewfd.murinvyz*ewfd.Ez))/mu0_const

ewfd.dHdtz → (ewfd.murinvzx*ewfd.dBdtx+ewfd.murinvzy*ewfd.dBdty+ewfd.murinvzz*ewfd.dBdtz-1i*ewfd.iomega/c_const*kappa*(ewfd.murinvzx*ewfd.Ex+ewfd.murinvzy*ewfd.Ey+ewfd.murinvzz*ewfd.Ez))/mu0_const

The optical constants of gold components were taken from the previous report by Johnson and Christy [4]. Considering that the range of 10^{-4} to 10^{-1} of chirality parameter κ is generally employed for computational simulations of concentrated molecular layers[5–9], a reasonable value of the chirality parameter κ in the range of 10^{-2} to 10^{-1} has been used for the simulation accordingly.

2. Supplementary Figures

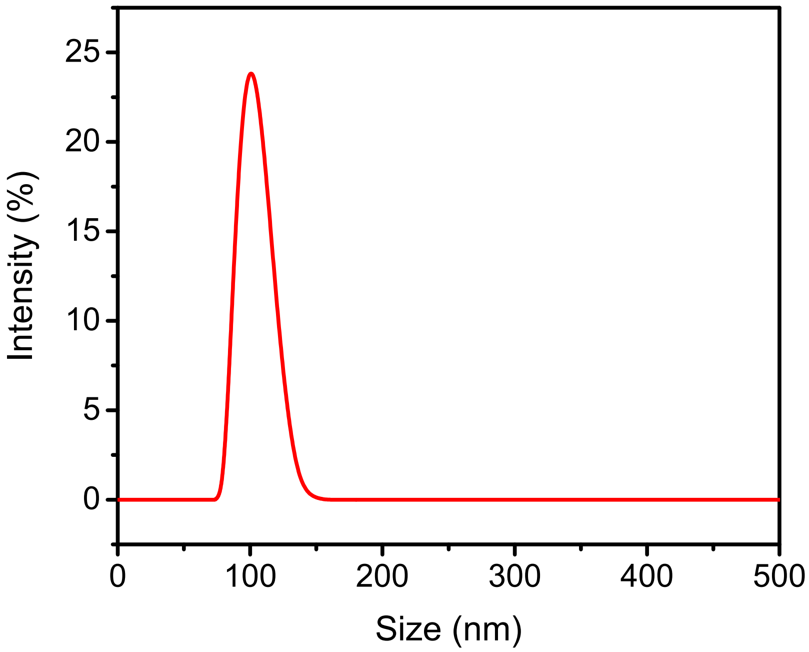

Figure S1 DLS measured distribution of gold helicoids size.

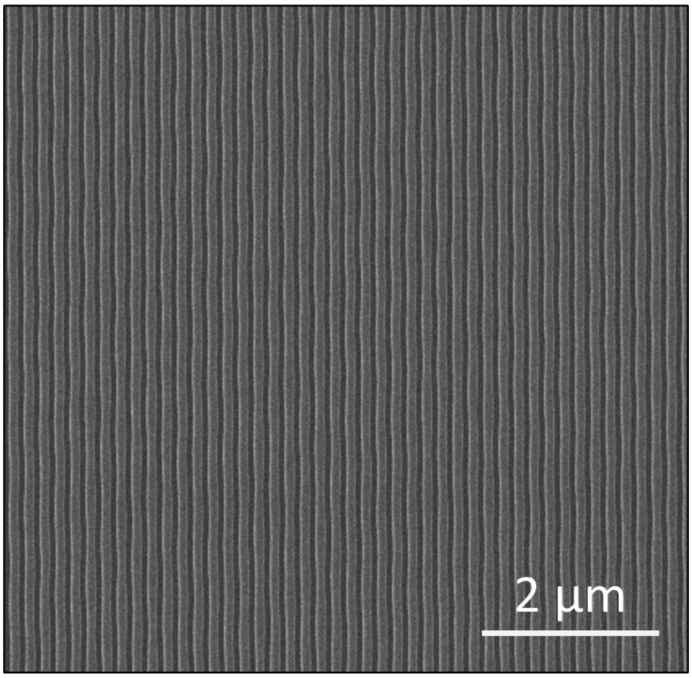

Figure S2 SEM image of the pristine gold grating.

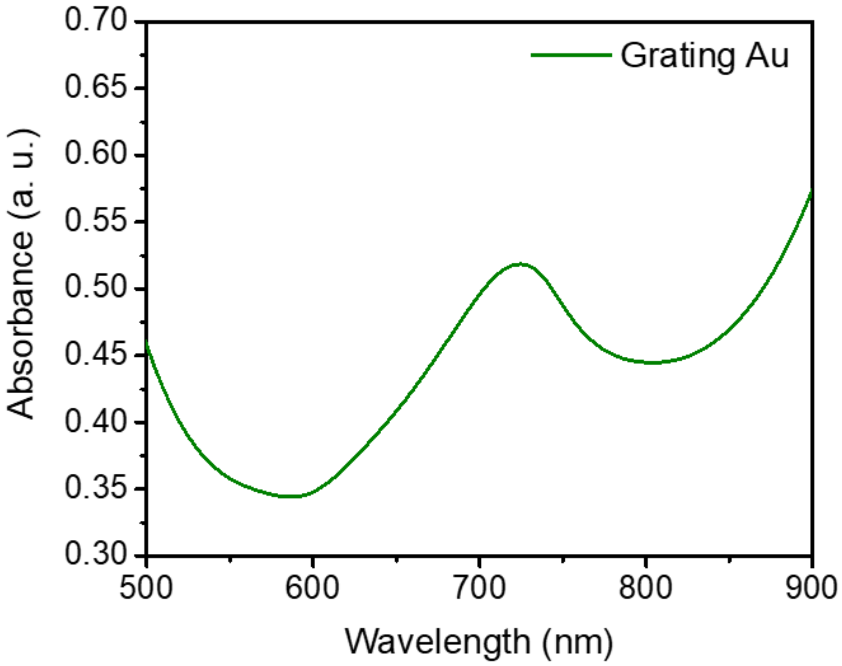

Figure S3 UV-Vis absorption spectrum of the pristine gold grating.

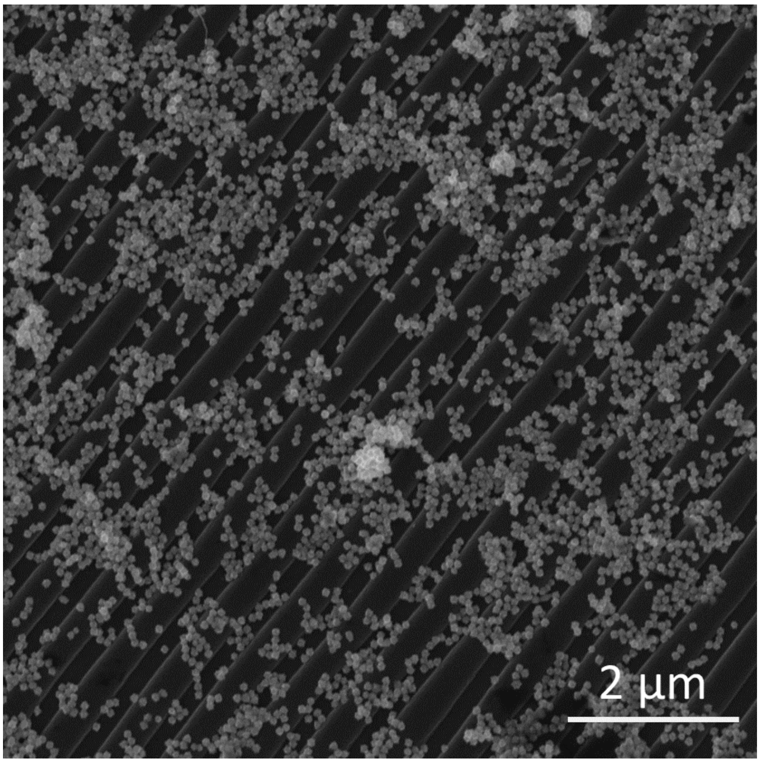

Figure S4 Example of unsuccessful distribution of helicoids on Au grating surface.

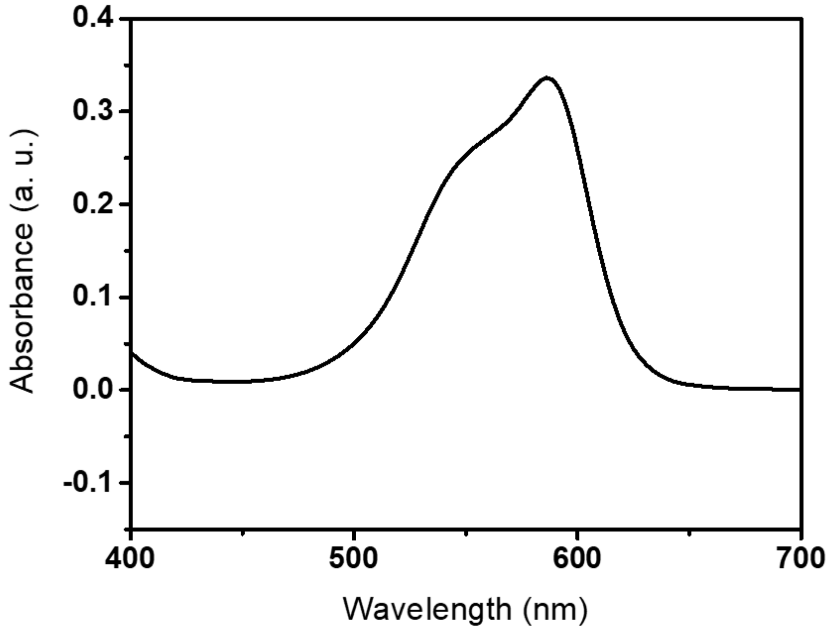

Figure S5 UV-Vis absorption spectra of crystal violet, drop deposited on glass substrate.

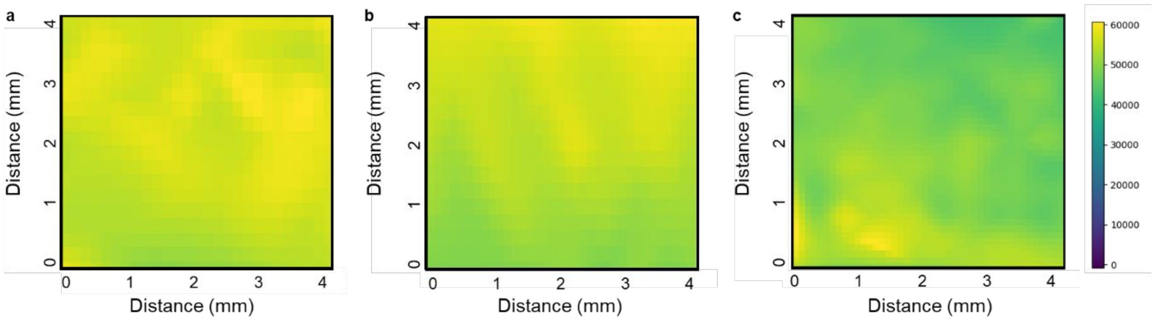

Figure S6 SERS map of crystal violet, measured on the surface of Au grating//helicoids surface (a, b, c – three different samples), excitation laser wavelength of 785 nm was used. The intensity and standard deviation of SERS peak (1148 cm^{-1}) are: 50112 ±1760, 51100 ± 2200, 48900 ± 4780 a.u (from left to right).

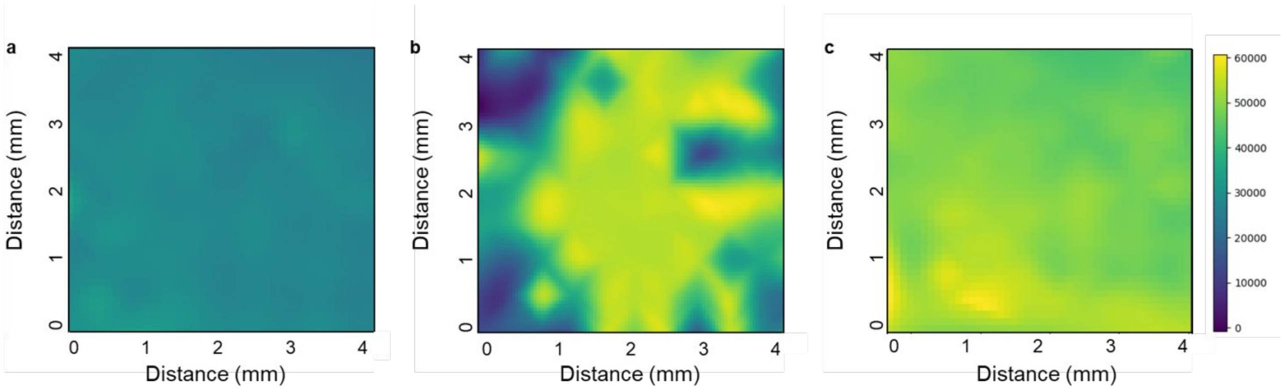

Figure S7 SERS maps of Crystal violet, measured **(a)** on the surface of Au grating, **(b)** helicoids and **(c)** Au grating/helicoids surface, 785 nm excitation laser wavelength was used.

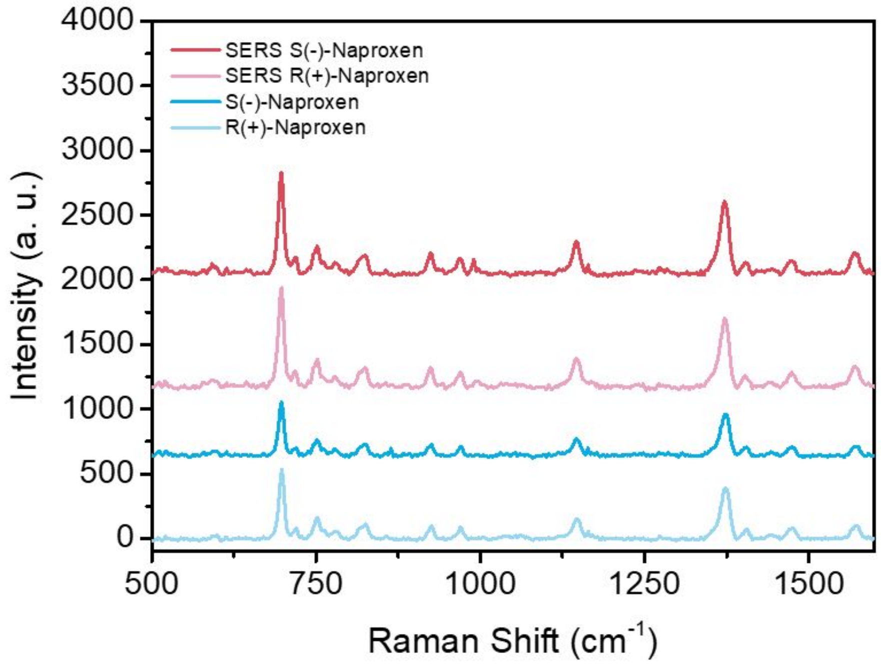

Figure S8 The Raman and SERS spectra (on Au grating without helicoids addition) recorded from pure R(+)-naproxen and S-(-)-naproxen.

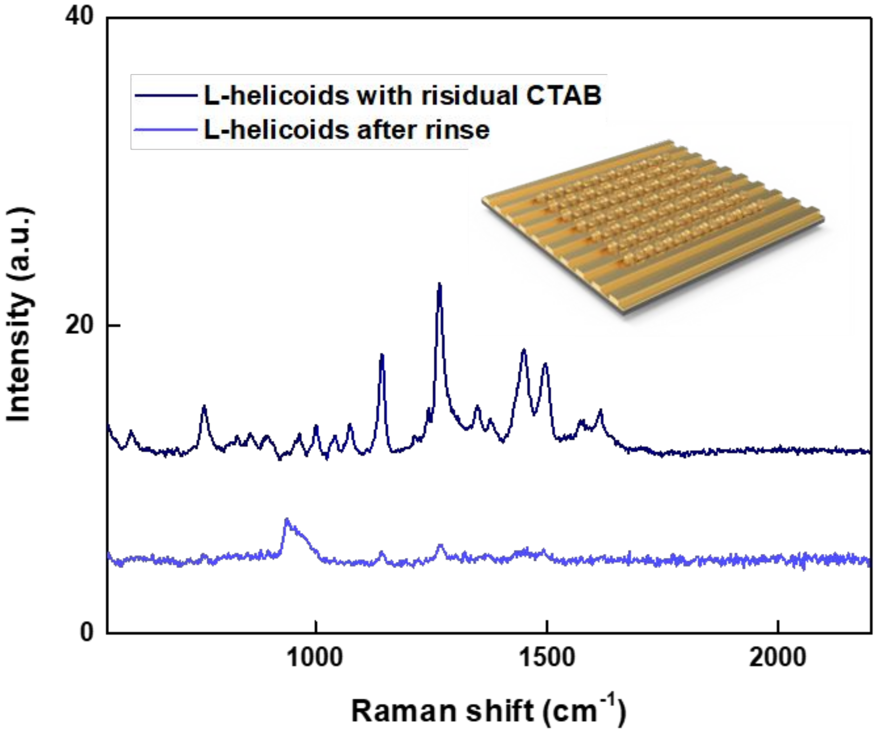

Figure S9 SERS spectra of L-helicoids, deposited on Au grating surface (without interaction of nanoparticles with naproxen molecules) before and after thorough rinse. SERS peaks are produced by residual CTAB, amount of which significantly decreased after the interaction of helicoids with methanol.

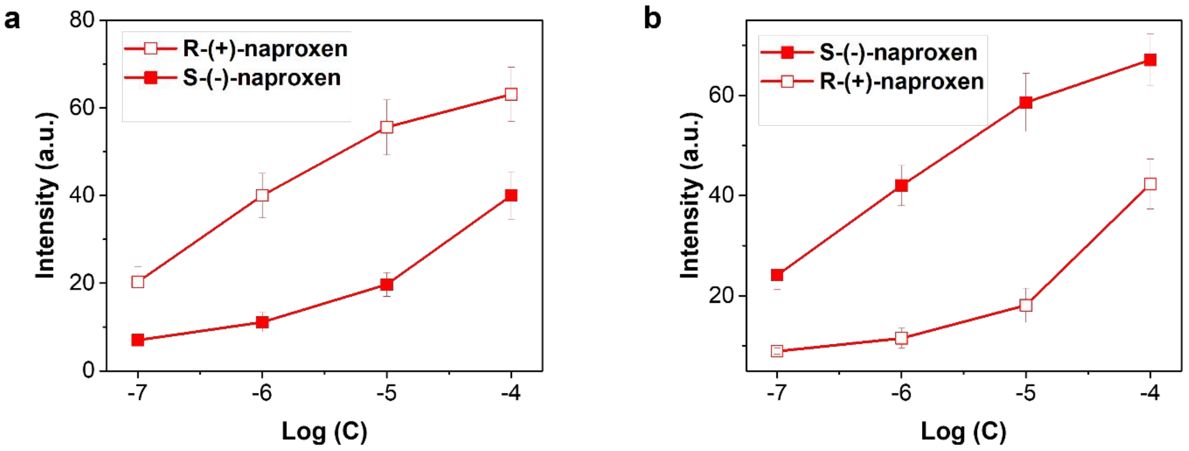

Figure S10 Characteristic SERS band intensity (1360 cm^{-1}) of R(+) or S(-) naproxen enantiomers as a function of initial naproxen enantiomers concentration (excitation wavelength – 785 nm): **(a)** L-helicoids//Au grating; **(b)** D-helicoids//Au grating substrates.

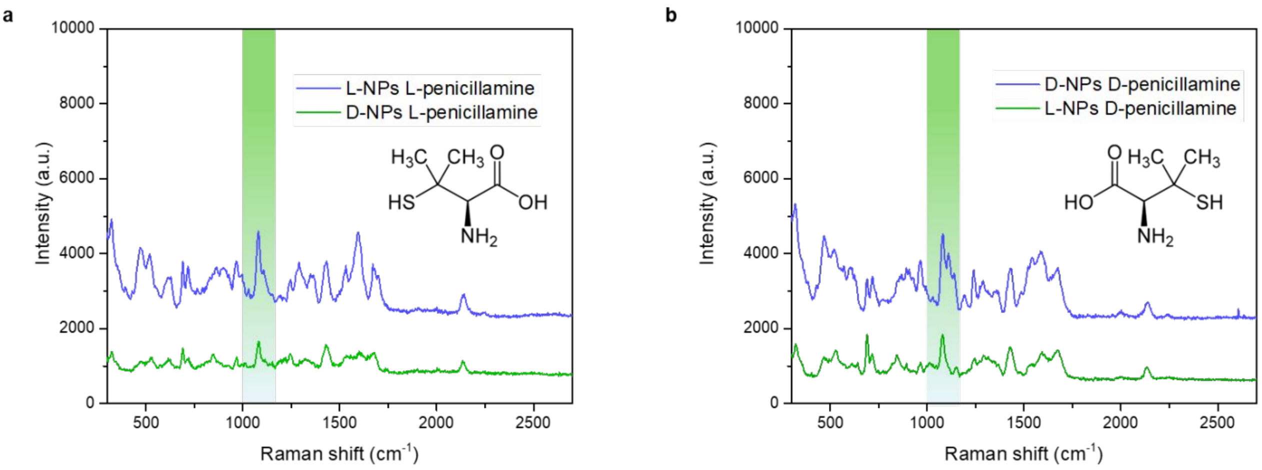

Figure S11 SERS spectra of L- or D-penicillamine enantiomers measured on Au helicoids/Au grating surface (drop deposition from 10^{-6} solution in MetOH). Spectra were measured at 50 different spots across macroscopic sample area – 5x5 mm, deviation between spectra was less than 4 %).

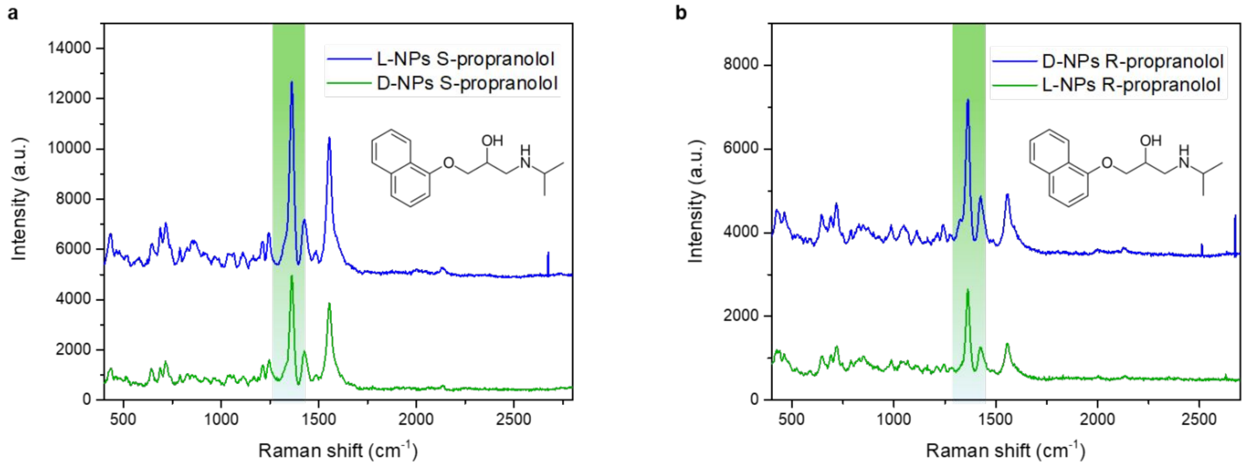

Figure S12 SERS spectra of R- or S-propranolol enantiomers measured on Au helicoids/Au grating surface (drop deposition from 10^{-6} solution in MetOH). Spectra were measured at 50 different spots across macroscopic sample area – 5x5 mm, deviation between spectra was less than 3,4 %).

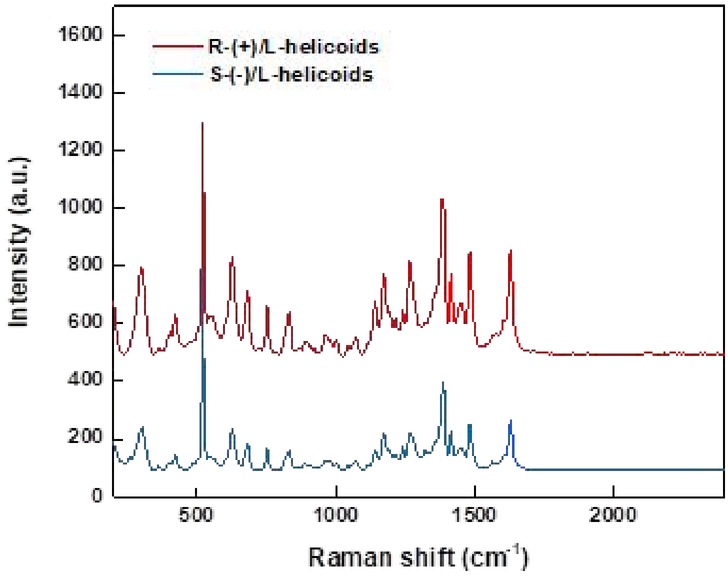

Figure S13 averaged SERS spectra of S(-) or R(+) naproxen enantiomers (deposited from 10^{-6} M solutions), measured on L-helicoids//Si substrates with the utilization of a 785 nm excitation wavelength.

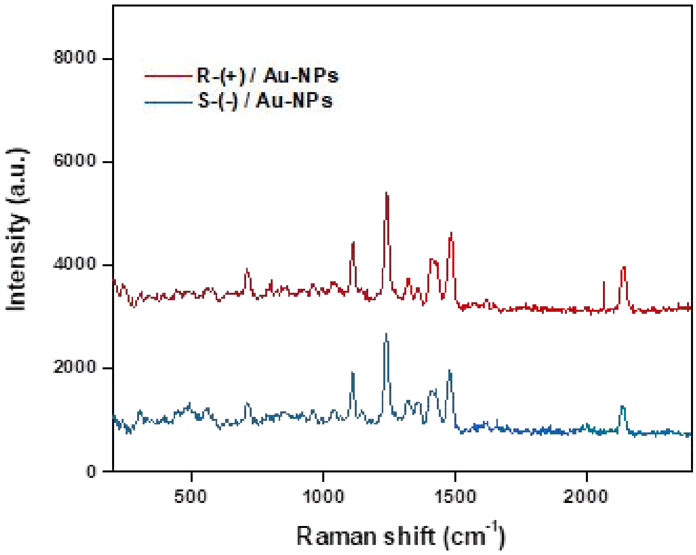

Figure S14 Averaged SERS spectra of R-(+) and S-(-) naproxene, measured with gold spherical NPs on the Au grating surface, (785 nm excitation laser wavelength, 30 spectra were measured at different spots and averaged for each case).

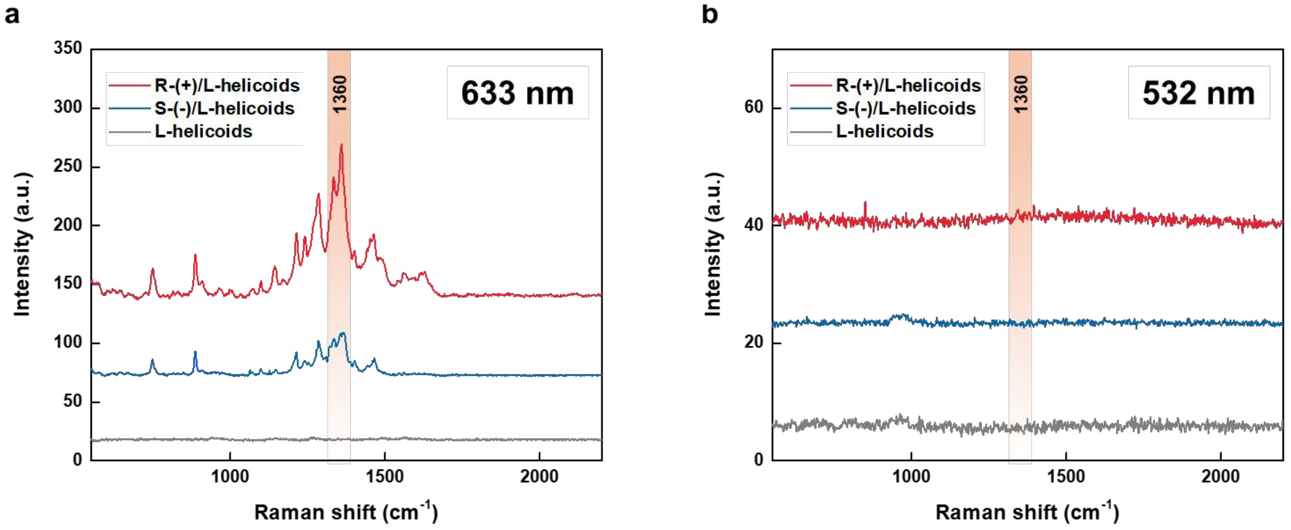

Figure S15 (a) - averaged SERS spectra of S(-) or R(+) naproxen enantiomers (deposited from 10^{-6} M solutions), measured on L-helicoids with the utilization of a 633 nm laser, **(b) -** averaged SERS spectra of S(-) or R(+) naproxen enantiomer, measured on L-helicoids with the utilization of a 532 nm laser.

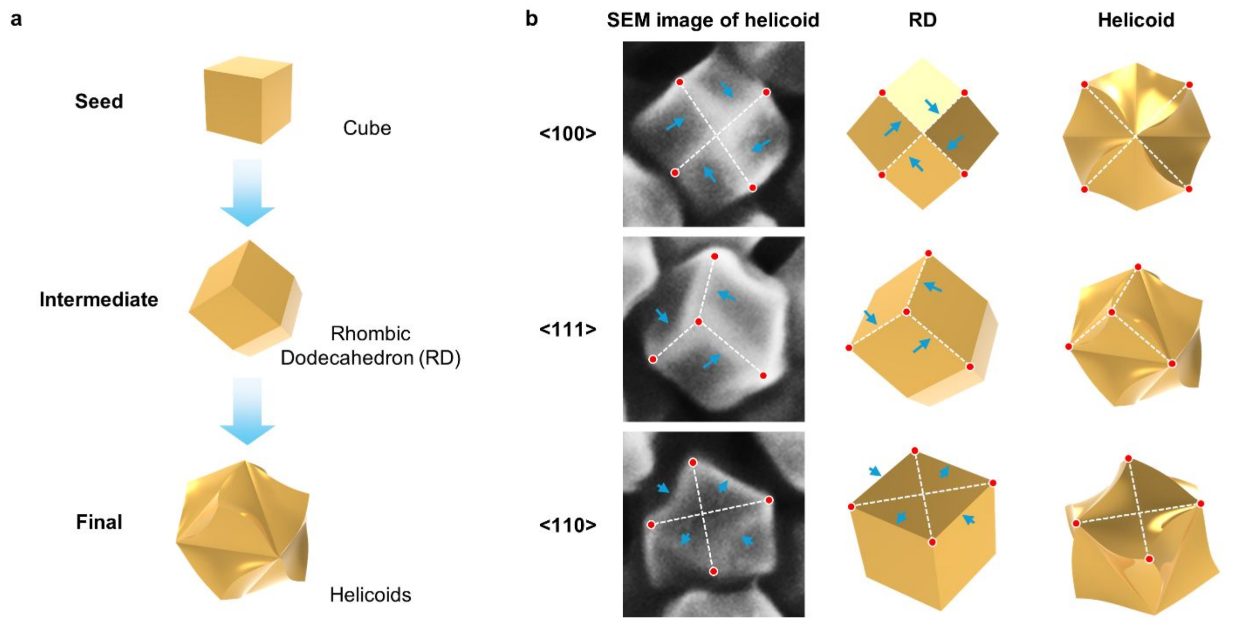

Figure S16 (a) Growth pathway of helicoids. The helicoid particles grow from seed cubic particles via the rhombic dodecahedron (RD) intermediate involving (100) and (110) planes. During the growth process involving chirality evolution, the basal shape remains that of the RD, with the particle volume increasing. **(b)** SEM images of the helicoids oriented along the <100>, <111>, <110> direction (left), 3D models of RD

(middle) and helicoid (right) particles. The 3D model of helicoid particles was constructed with the RD as the base shape, with the edges twisted referring to the SEM image.

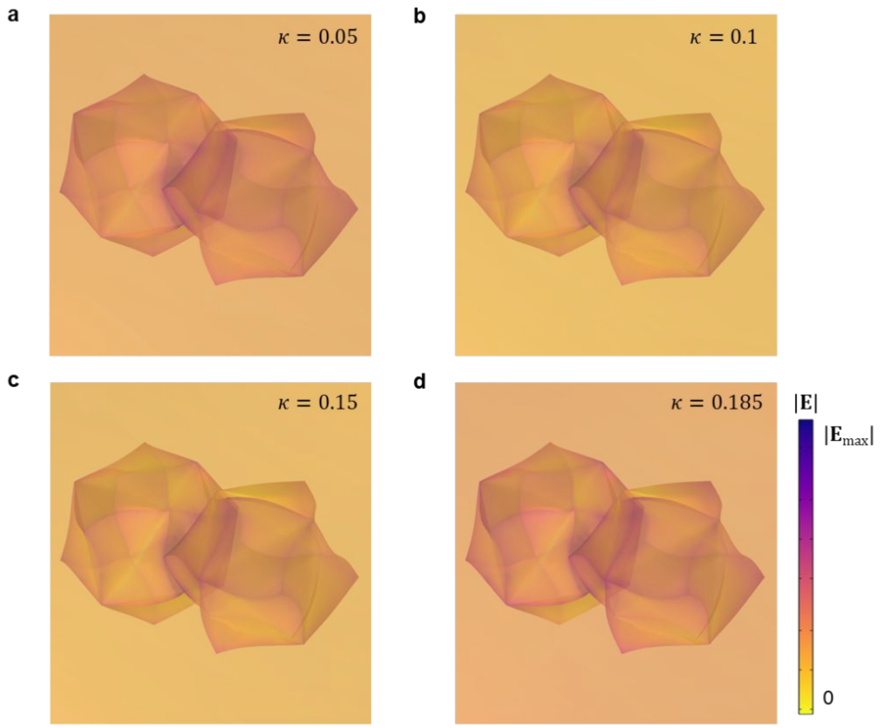

Figure S17 (a) to **(d) -** electric field intensity in the vicinity of a L-helicoid dimer for two values of the chirality parameter κ of surrounding medium. The higher the field intensity outside the nanogap (i.e., surrounding media and nanoparticle surfaces), the lower is the enantioselectivity calculated based on the SERS enhancement factor.

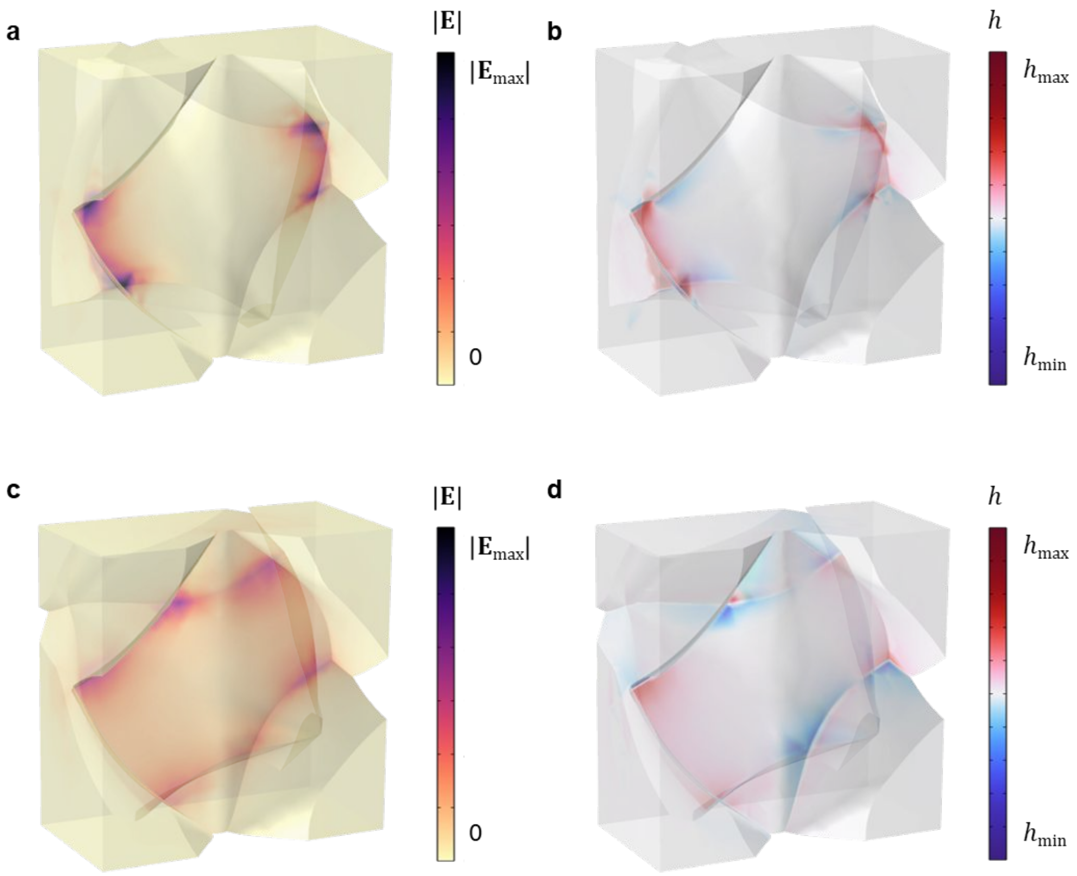

Figure S18 Spatial profiles of: **(a) -** electric field ($|\mathbf{E}|^2$) and **(b) -** optical helicity density h at the nanogap of L-helicoid dimer for θ=30 degrees. Spatial profiles of: **(c) -** electric field ($|\mathbf{E}|^2$) and **(d) -** optical helicity density h for θ=60 degrees. Strong optical helicity density h with uniform sign (positive) is induced over the electric field hotspots.

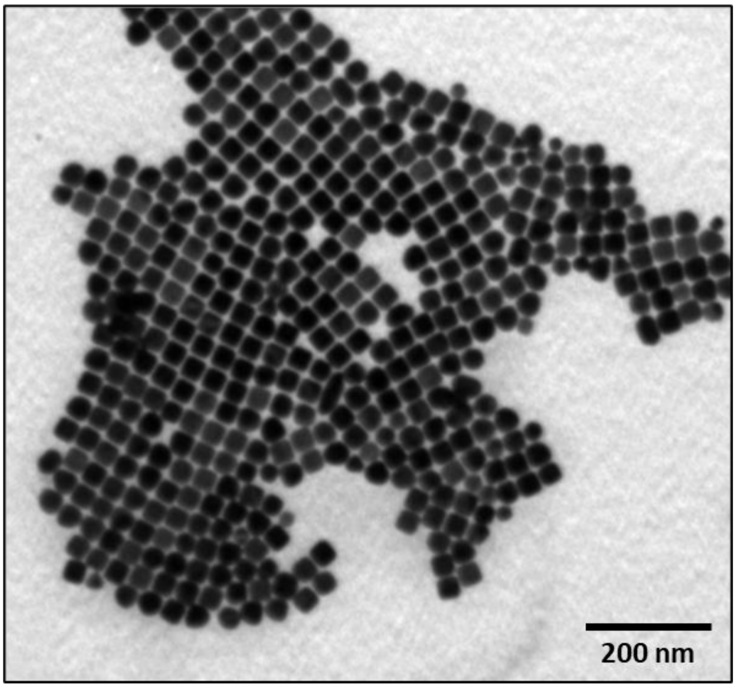

Figure S19 Characterization of Au nanocubes, used for helicoids preparation (TEM).

References:

(1) Mun, J.; Rho, J. Importance of Higher-Order Multipole Transitions on Chiral Nearfield Interactions. *Nanophotonics* **2019**, *8* (5), 941–948. https://doi.org/10.1515/nanoph-2019-0046.

(2) Nesterov, M. L.; Yin, X.; Schäferling, M.; Giessen, H.; Weiss, T. The Role of Plasmon-Generated Near Fields for Enhanced Circular Dichroism Spectroscopy. *ACS Photonics* **2016**, *3* (4), 578–583. https://doi.org/10.1021/acsphotonics.5b00637.

(3) Kelly, C.; Khosravi Khorashad, L.; Gadegaard, N.; Barron, L. D.; Govorov, A. O.; Karimullah, A. S.; Kadodwala, M. Controlling Metamaterial Transparency with Superchiral Fields. *ACS Photonics* **2018**, *5* (2), 535–543. https://doi.org/10.1021/acsphotonics.7b01071.

(4) Johnson, P. B.; Christy, R. W. Optical Constants of the Noble Metals. *Phys. Rev. B* **1972**, *6* (12), 4370–4379. https://doi.org/10.1103/PhysRevB.6.4370.

(5) García-Guirado, J.; Svedendahl, M.; Puigdollers, J.; Quidant, R. Enhanced Chiral Sensing with Dielectric Nanoresonators. *Nano Lett.* **2020**, *20* (1), 585–591. https://doi.org/10.1021/acs.nanolett.9b04334.

(6) Abdulrahman, N. A.; Fan, Z.; Tonooka, T.; Kelly, S. M.; Gadegaard, N.; Hendry, E.; Govorov, A. O.; Kadodwala, M. Induced Chirality through Electromagnetic Coupling between Chiral Molecular Layers and Plasmonic Nanostructures. *Nano Lett.* **2012**, *12* (2), 977–983. https://doi.org/10.1021/nl204055r.

(7) Lee, S.; Kang, J.-H.; Yoo, S.; Park, Q.-H. Robust Numerical Evaluation of Circular Dichroism from Chiral Medium/Nanostructure Coupled Systems Using the Finite-Element Method. *Sci. Rep.* **2018**, *8* (1), 8406. https://doi.org/10.1038/s41598-018-26815-5.

(8) Mohammadi, E.; Tsakmakidis, K. L.; Askarpour, A. N.; Dehkhoda, P.; Tavakoli, A.; Altug, H. Nanophotonic Platforms for Enhanced Chiral Sensing. *ACS Photonics* **2018**, *5* (7), 2669–2675. https://doi.org/10.1021/acsphotonics.8b00270.

(9) Han, J. H.; Lim, Y.-C.; Kim, R. M.; Lv, J.; Cho, N. H.; Kim, H.; Namgung, S. D.; Im, S. W.; Nam, K. T. Neural-Network-Enabled Design of a Chiral Plasmonic Nanodimer for Target-Specific Chirality Sensing. *ACS Nano* **2023**, *17* (3), 2306–2317. https://doi.org/10.1021/acsnano.2c08867.
